# Knowledge and Attitudes Concerning Aducanumab Among Older Americans After FDA Approval for Treatment of Alzheimer Disease

**DOI:** 10.1001/jamanetworkopen.2021.48355

**Published:** 2022-02-14

**Authors:** Julie Zissimopoulos, Mireille Jacobson, Yi Chen, Soo Borson

**Affiliations:** 1 Department of Health Policy and Management, Sol Price School of Public Policy, University of Southern California, Los Angeles; 2 Schaeffer Center for Health Policy and Economics, University of Southern California, Los Angeles; 3 Andrus School of Gerontology, University of Southern California, Los Angeles; 4 Sol Price School of Public Policy, University of Southern California, Los Angeles; 5 Department of Family Medicine, Keck School of Medicine, University of Southern California, Los Angeles

## Abstract

This survey study examines the understanding of aducanumab and attitudes toward its potential outcomes among older Americans after the drug's approval by the US Food and Drug Administration (FDA) for the treatment for Alzheimer disease.

## Introduction

On June 7, 2021, the Food and Drug Administration (FDA) approved aducanumab (Aduhelm), the first new drug for Alzheimer disease in decades. Response to the decision among clinical and research communities was immediate.^1,2,3,4^ Enthusiasm was outweighed by concerns that included uncertain efficacy, serious adverse effects, a broad target patient population, high costs, and potential association with demand for diagnostics.

While the Centers for Medicare & Medicaid Services has proposed a restrictive coverage decision, patients and their doctors may still choose aducanumab. Informed use decisions should weigh potential benefits and harms associated with the drug, which presumes that individuals understand these issues. Soon after aducanumab’s approval, when publicity was high, we studied older Americans’ understanding of aducanumab and attitudes toward its potential outcomes.

## Methods

This survey study collected data from individuals in the Understanding America Study (UAS). The University of Southern California’s Institutional Review Board (IRB) approved the study as an amendment to UP-14-00148. UAS obtained written informed consent from participants. The UAS IRB approval and informed consent requirement extended to this survey study, which followed the American Association for Public Opinion Research (AAPOR) reporting guideline.

UAS recruits individuals ages 18 years and older from a nationally representative sampling frame, which was also used in this survey study.^5^ People without online access are provided a tablet and internet subscription. Between July 15 and August 11, 2021, we conducted an online survey about aducanumab knowledge and attitudes toward its potential outcomes among 1333 UAS participants ages 55 years and older, and 1035 individuals responded (response rate = 77.5%). Respondents were paid $2 to complete the survey.

We measured self-assessed knowledge about aducanumab and the number of correct responses to 6 questions about the drug, and we used scales of agreement to characterize attitudes toward outcomes associated with aducanumab (eAppendix in the Supplement). Using logit regression models, we estimated associations of respondent sex, age, self-reported race and ethnicity, and education with 2 outcomes: odds of seeking cognitive screening after drug approval and demand for aducanumab if diagnosed with Alzheimer disease. Survey response options for race included African American, American Indian or Alaska Native, Asian American, Native Hawaiian or other Pacific Islander, and Caucasian (hereafter, *non-Hispanic White*), and respondents could choose 2 or more races. For ethnicity, respondents could choose yes or no for the option of Hispanic or Latino. Race and ethnicity data were collected because Alzheimer disease risk is increased for Black and Hispanic individuals compared with White individuals, thus leading potential heterogeneity across racial groups in the association of race and ethnicity with this study's outcomes. Statistical analyses used Stata statistical software version 16.1 (StataCorp). Statistical significance was set at *P* < .05, and *P* values were 2-tailed. Data were analyzed from August through September 2021.

## Results

Among 1035 respondents, mean (SD) age was 67 (7.9) years, and there were 567 (54.8%) women. There were 67 African American individuals (6.5%), 21 Asian American individuals (2.0%), 7 American Indian or Alaska Native individuals (0.7%), 2 Native Hawaiian or other Pacific Islander individuals (0.2%), 865 non-Hispanic White individuals (83.6%), and 37 individuals with 2 or more races (3.6%); there were 36 Hispanic or Latino individuals (3.5%). There were 404 individuals (39.0%) with a bachelor’s or higher degree. Despite concern about Alzheimer disease among 878 of 1035 individuals who saw and responded to the question (84.8%), 277 individuals (26.8%) reported some or fair amount of knowledge of aducanumab. The number of correct responses to 6 true or false questions about efficacy, patient population, administration procedure, adverse effects, costs, and FDA expert panel endorsement was 0 at the median, 1 at the 75th percentile, and 3 at the 90th percentile (Table). Among 1030 respondents who saw and answered the question, 451 individuals (43.8%) agreed that aducanumab would provide a societal benefit. Approximately half were concerned about costs to individuals (504 of 1030 individuals who saw and answered the question [48.9%]) and Medicare (464 of 1030 individuals who saw and answered the question [45.0%]). Nearly two-thirds of 977 respondents who saw and answered the question (590 individuals [60.4%]) were uncertain about the association of aducanumab with their odds of seeking cognitive impairment screening. Among 977 individuals with nonmissing values, Non-Hispanic White individuals were less likely to report increased chance of screening than individuals from other racial and ethnic groups (odds ratio [OR], 0.62; 95% CI, 0.42-0.90; *P* = .01) (Figure, A). Other racial and ethnic groups were grouped together for this analysis owing to small sample size and included African American, Asian American, American Indian or Alaska Native, Native Hawaiian or other Pacific Islander, and Hispanic or Latino individuals and individuals reporting 2 or more races. Among 1033 respondents who saw and answered the question, there were 240 individuals (23.2%) who said they would want to receive aducanumab if they had Alzheimer disease. Individuals ages 60 to 75 years were less likely than those ages 55 to 59 years to respond affirmatively to wanting aducanumab treatment (60-64 years: OR, 0.61; 95% CI, 0.39-0.94; *P* = .03; 65 to 70 years: OR, 0.64; 95% CI, 0.42-0.97; *P* = .04; 71-75 years: OR, 0.55; 95% CI, 0.34-0.90; *P* = .02) (Figure, B).

**Table.** Knowledge of Aducanumab and Attitude Toward Outcomes

Survey question	Respondents, No. (%) (N = 1035)^a^
Concern about Alzheimer disease (n = 1035)^b^	
Very concerned	456 (44.1)
Somewhat concerned	422 (40.8)
A little or not at all concerned	157 (15.2)
At least some knowledge about aducanumab, self-assessed (n = 1035)	277 (26.8)
No. of correct responses to 6 true or false questions about aducanumab^c^	
Mean (SD)	0.76 (1.34)
Median (75th percentile to 90th percentile)	0 (1-3)
Agree or disagree with statements about aducanumab^d^	
Will provide important benefits to society (n = 1030)	
Agree	451 (43.8)
Neither agree nor disagree	519 (50.4)
Disagree	60 (5.8)
Is a major breakthrough in Alzheimer disease treatment (n = 1031)	
Agree	430 (41.7)
Neither agree nor disagree	531 (51.5)
Disagree	70 (6.8)
Will be costly to Medicare (n = 1030)^b^	
Agree	464 (45.0)
Neither agree nor disagree	534 (51.8)
Disagree	32 (3.1)
Will be expensive for patients (n = 1030)	
Agree	504 (48.9)
Neither agree nor disagree	509 (49.4)
Disagree	17 (1.7)

^a^ Percentages and distribution of number of correct responses to true or false questions are based on all survey participants. Population numbers for each survey question are individuals who saw and responded to the question.

^b^ Percentages do not sum to 100% owing to rounding.

^c^ The 6 true or false questions about aducanumab are described in the eAppendix in the Supplement.

^d^ Agree and disagree include answers of strongly agree and somewhat agree.

**Figure.**
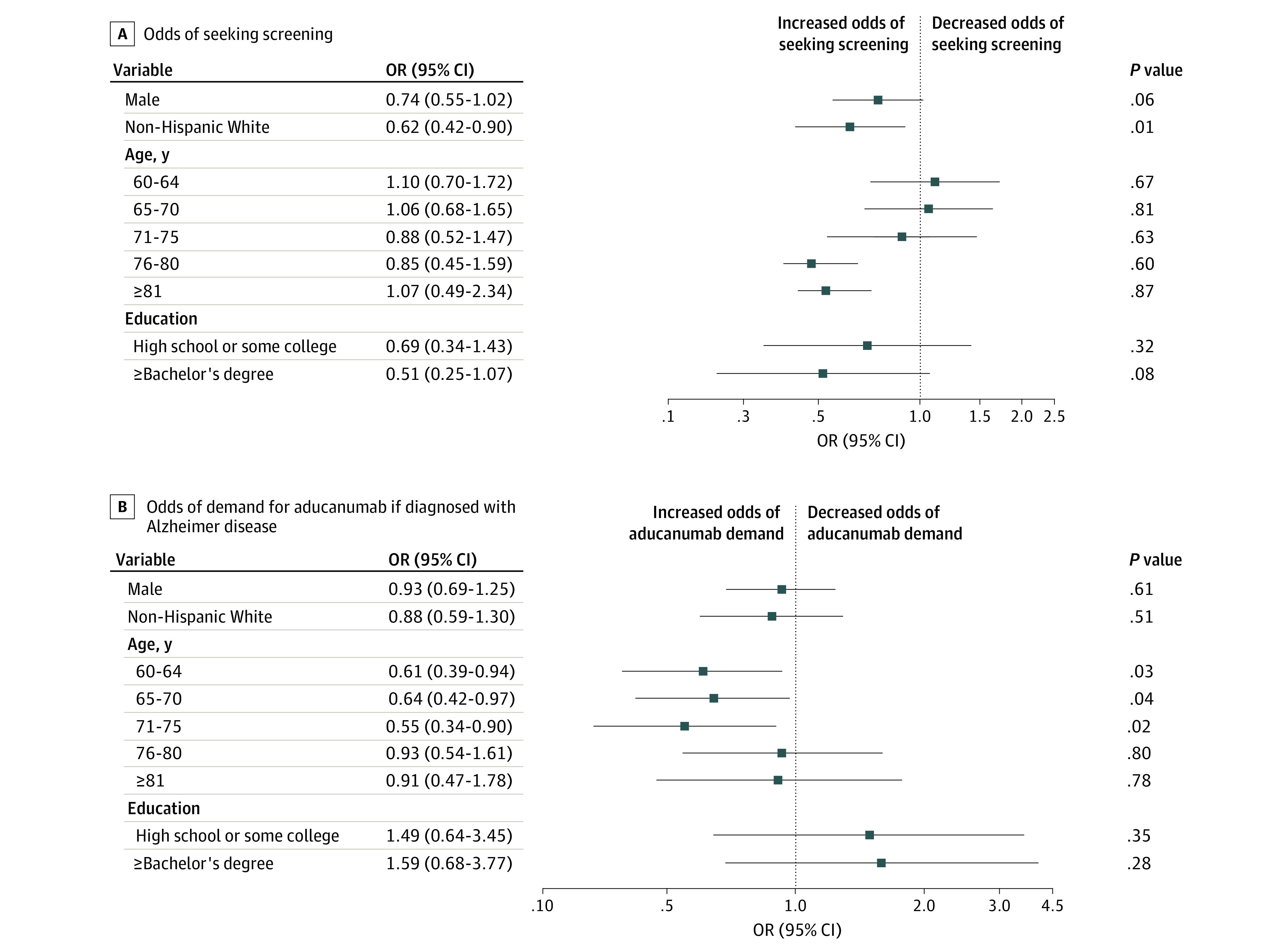
Odds of Agreement With Statements About Aducanumab Odds ratios (ORs) and 95% CIs for agreeing with the statements “increases chances will seek screening” and “would want to receive Aduhelm (aducanumab) if had Alzheimer disease” are shown in A and B, respectively. ORs were obtained from multivariate logistic regressions adjusting for sex, age group, race and ethnicity, and education. Ages 55 to 59 years is the reference group for other age groups, and less than high school is the reference group for other education groups. The reference group for non-Hispanic White individuals was members of other race and ethnicity groups, which included African American, Asian American, American Indian or Alaska Native, Hispanic or Latino, and Native Hawaiian or other Pacific Islander individuals and individuals reporting 2 or more races. The sample included 977 individuals who saw and answered question A and 1033 individuals who saw and answered question B.

## Discussion

This survey study found that the widespread and contentious publicity after FDA approval of aducanumab in June 2021 was not associated with a broad understanding of nor enthusiasm for this new therapeutic agent among middle-aged and older adults. Major specialty organizations, including the American Academy of Neurology,^6^ issued patient-directed and clinician-directed information sheets about treating Alzheimer disease with aducanumab. This study’s results suggest a need for information sharing and processes for guiding decision-making of potential patients and their prescribing partners. Study limitations included inadequate sample size to assess heterogeneity across other characteristics and a cross-sectional design, which precluded study of survey changes in knowledge or attitudes.

With FDA approval of aducanumab and more drugs in the pipeline, treatment options for Alzheimer disease are expanding. Health care professionals will need to educate patients about potential costs and benefits and to support patients’ decision making.

## Notes

Supplement. **eAppendix.** Understanding America Study Survey Questions About Aducanumab
