# Supplementary material for: Knowledge and Attitudes Concerning Aducanumab Among Older Americans After FDA Approval for Treatment of Alzheimer Disease
Source: JAMA Netw Open. 2022 Feb 14;5(2):e2148355. doi: 10.1001/jamanetworkopen.2021.48355 (PMC8845005; doi:10.1001/jamanetworkopen.2021.48355)
Supplement: Supplement. — eAppendix. Understanding America Study Survey Questions About Aducanumab [file jamanetwopen-e2148355-s001.pdf]

Supplemental Online Content

Zissimopoulos J, Jacobson M, Chen Y, Borson S. Knowledge and attitudes concerning aducanumab among older Americans after FDA approval for treatment of Alzheimer disease. *JAMA Netw Open*. 2022;5(2):e2148355.
doi:10.1001/jamanetworkopen.2021.48355

eAppendix. Understanding America Study Survey Questions About Aducanumab

This supplemental material has been provided by the authors to give readers additional information about their work.

© 2022 Zissimopoulos J et al. *JAMA Network Open.*

eAppendix. Understanding America Study Survey Questions About Aducanumab

Answers to true/false questions are shown in *italic*.

On June 7, 2021, the Food and Drug Administration (FDA) approved Aduhelm (previously known as aducanumab), the first new drug for Alzheimer's disease in almost 20 years. Aducanumab is a medication that may help slow the progression of early stage Alzheimer's disease. Alzheimer's disease is a longterm, progressive brain disorder that slowly destroys memory and thinking skills and, eventually, the ability to carry out simple tasks.

1. How much would you say you know about this new drug treatment for Alzheimer's, Aduhelm (aducanumab) ?

(1) A fair amount
(2) Just some
(3) Nothing

2. How concerned are you about the risk of Alzheimer's disease for Americans and their families?
(1) Very concerned
(2) Somewhat concerned
(3) Just a little concerned
(4) Not at all concerned

3. The scientific evidence on the efficacy of this drug for slowing the progression of Alzheimer's disease is strong
(1) True
(2) *False*
(3) Don't know

4. Only patients with moderate or severe Alzheimer's disease are recommended to use Aduhelm (aducanumab)
(1) True
(2) *False*
(3) Don't know

5. Aduhelm (aducanumab) will be administered by infusion into a vein.
(1) *True*
(2) False
(3) Don't know

6. Aduhelm (aducanumab) has side effects including headache, confusion, dizziness, vision changes, or nausea.
(1) *True*
(2) False
(3) Don't know

7. The price of Aduhelm (aducanumab) will be $56,000 per patient per year

© 2022 Zissimopoulos J et al. *JAMA Network Open.*

(1) *True*
(2) False
(3) Don’t know

8. Medicare and other health insurers will likely pay for the cost of Aduhelm (aducanumab) less any copayments, deductibles. (authors: excluded from analysis of knowledge)
(1) *True*
(2) False
(3) Don’t know

9. The FDA’s expert panel strongly endorsed approval of Aduhelm (aducanumab).
(1) True
(2) *False*
(3) Don’t know

10. Do you are or disagree with the following statements about Aduhelm (aducanumab):

	Strongly agree	Somewhat agree	Neither agree nor disagree	Somewhat disagree	Strongly disagree
Will provide important benefits to society					
Is a major breakthrough in Alzheimer’s disease treatment					
Will be costly to Medicare					
Will be expensive for patients					
Has serious side effects					
Increases the chance I will seek screening or testing for cognitive impairment					

11. I would want to receive Aduhelm if I had Alzheimer’s disease
(1) Yes
(2) No
(3) Uncertain

IF Respondent says **NO or uncertain**:

12. You said you (**fill here** were uncertain or would not want to) receive the drug treatment Aduhelm if you had Alzheimer’s disease. Please check the reasons why you made that choice.
(1) Drug will not be effective
(2) Concerned about side effects
(3) Concerned about cost of the treatment

© 2022 Zissimopoulos J et al. *JAMA Network Open.*
